# [Retracted] MicroRNA-18a-5p represses scar fibroblast proliferation and extracellular matrix deposition through regulating Smad2 expression

**DOI:** 10.3892/etm.2026.13139

**Published:** 2026-03-23

**Authors:** Tianshi Li, Yiguang Wu, Dandan Liu, Lida Zhuang

Exp Ther Med 22:1318, 2021; DOI: 10.3892/etm.2021.10753

Following the publication of this paper, it was drawn to the Editor's attention by a concerned reader that flow cytometric (FCM) assay data featured in Figs. 3C and 5D were strikingly similar to FCM data which were ultimately published in a number of other papers in different journals that were written by different authors at different research institutes, several of which had already been published before this article was received at *Experimental and Therapeutic Medicine.*

Owing to the fact that the contentious data in the above article were found to be strikingly similar to data that had already been published elsewhere in other papers in different journals, the Editor of *Experimental and Therapeutic Medicine* has decided that this paper should be retracted from the Journal. The authors were asked for an explanation to account for these concerns, but the Editorial Office did not receive a reply. The Editor apologizes to the readership for any inconvenience caused.

